# System analysis based on the ER stress-related genes identifies WFS1 as a novel therapy target for colon cancer

**DOI:** 10.18632/aging.204404

**Published:** 2022-11-28

**Authors:** Xianguang Yang, Chaoyang Zhang, Cheng Yan, Liukai Ma, Jiahao Ma, Xiaoke Meng

**Affiliations:** 1 School of Life Sciences, State Key Laboratory Base of Cell Differentiation and Regulation, Henan Normal University, Xinxiang 453007, China; 2 School of Pharmacy, Key Laboratory of Nano-carbon Modified Film Technology of Henan Province, Diagnostic Laboratory of Animal Diseases, Xinxiang University, Xinxiang, Henan 453000, China

**Keywords:** COAD, ERSRGs, prognostic models, sensitive drugs, WFS1

## Abstract

Background: Colon cancer (COAD) is the third-largest common malignant tumor and the fourth major cause of cancer death in the world. Endoplasmic reticulum (ER) stress has a great influence on cell growth, migration, proliferation, invasion, angiogenesis, and chemoresistance of massive tumors. Although ER stress is known to play an important role in various types of cancer, the prognostic model based on ER stress-related genes (ERSRGs) in colon cancer has not been constructed yet. In this study, we established an ERSRGs prognostic risk model to assess the survival of COAD patients.

Methods: The COAD gene expression profile and clinical information data of the training set were obtained from the GEO database (GSE40967) and the test set COAD gene expression profile and clinical informative data were downloaded from the TCGA database. The endoplasmic reticulum stress-related genes (ERSRGs) were obtained from Gene Set Enrichment Analysis (GSEA) website. Differentially expressed ERSRGs between normal samples and COAD samples were identified by R “limma” package. Based on the univariate, lasso, and multivariate Cox regression analysis, we developed an ERSRGs prognostic risk model to predict survival in COAD patients. Finally, we verified the function of WFS1 in COAD through *in vitro* experiments.

Results: We built a 9-gene prognostic risk model based on the univariate, lasso, and multivariate Cox regression analysis. Kaplan-Meier survival analysis and Receiver operating characteristic (ROC) curve revealed that the prognostic risk model has good predictive performance. Subsequently, we screened 60 compounds with significant differences in the estimated half-maximal inhibitory concentration (IC50) between high-risk and low-risk groups. In addition, we found that the ERSRGs prognostic risk model was related to immune cell infiltration and the expression of immune checkpoint molecules. Finally, we determined that knockdown of the expression of WFS1 inhibits the proliferation of colon cancer cells.

Conclusions: The prognostic risk model we built may help clinicians accurately predict the survival of patients with COAD. Our findings provide valuable insights into the role of ERSRGs in COAD and may provide new targets for COAD therapy.

## INTRODUCTION

Colon cancer (COAD) is the third-largest commonly diagnosed tumor and the fourth major cause of cancer-associated mortality in the world [1]. Early COAD patients may be successfully cured by surgery. However, most advanced patients of COAD have experienced recurrence and metastases, and their five-year survival rate is usually no more than 10% [2]. Owing to the development in surgical techniques, the mortality rate of COAD patients has significantly decreased. However, COAD patients still face poor prognosis because of increased postoperative complications and drug resistance [3, 4].

ER is one of the utmost organelles in cells and that is the main place for protein biosynthesis and folding, lipid and steroid hormones production, glucose metabolism, and calcium release [5]. Normal cellular function relies heavily on ER homeostasis. ER stress occurs in many conditions, such as oxidative stress, nutritional deficiency, accumulation of mutant proteins, hypoxia and metabolic stress, virus, and loss of calcium homeostasis [6, 7]. In response to ER stress, cells will initiate an adaptive reaction to solve ER stress and restore cell homeostasis, which is called unfolded protein response (UPR) [8]. Growing evidence indicates that ER stress and UPR perform vital functions during the progression of multiple tumors, such as colon cancer, hepatocellular carcinoma, and glioma [9, 10]. In addition, increased UPR activity affects a number of intracellular metabolic pathways, which ultimately shape the tumor microenvironment [11]. Thus, treatment strategies targeting components of the UPR and reducing ER stress will be promising therapeutic approaches. To our knowledge, the prognostic model relied on ER Stress-Related genes (ERSRGs) for COAD has not been reported.

Herein, we built a prognostic model relied on ERSRGs for COAD and validated it in an external dataset. Afterwards, we explored the relationship between the ERSRGs signature and infiltration of immune cell in immune microenvironment. At last, we investigated the role of WFS1 in COAD through *in vitro* experiments.

## METHODS

### Data collection

A total of 419 ER stress-associated genes are derived from the Gene Set Enrichment Analysis (GSEA) website. Gene expression profiles of GSE40967 (training set) and its corresponding clinical characteristics are downloaded from the gene expression omnibus, containing 19 normal samples and 566 tumor samples. Furthermore, gene expression profiles of 41 samples from normal colon tissue and 473 samples from tumor specimens are derived from The Cancer Genome Atlas (TCGA) database (Downloaded 2022.01.01), which is used as validation set.

### Removing batch effect

We adjusted the test set to the training set using R “limma” package and R “sva” package.

### Identifying differentially expressed genes (DEGs)

We detected DEGs between the tumor and normal tissues by R “limma” package. False discovery rate (FDR) <0.05 and |log_2_foldchange| >1 were set as the cutoff criteria. Then, heatmap was plotted with R heatmap package. Protein-protein interaction (PPI) network was built via STRING database. Cyto-hubba plug-in from Cytoscape software was performed to detect hub genes.

### Developing and validating a prognostic model relied on ERSRGs

To determine which genes were associated with death, univariate Cox regression analyses were conducted using the training set. Candidates for ERSRGs associated with overall survival were those with *p*-value <0.05. By performing lasso regression, we were able to reduce the model complexity and multicollinearity by shrinking the coefficients. For the purpose of developing a survival model, we conducted multivariate Cox regression analyses. Applying the formula below, we calculated the risk scores for each patient:


$$\text{risk score}=\sum_{j=1}^{n}\left(Coef_{j}\times Xj\right)$$

*Xj* measures gene expression value of genes, and *Coefj* represents the regression coefficient. High-risk and low-risk patients were grouped based on median scores calculated by the risk score formula. We compared the overall survival gaps between high-risk and low-risk groups using the Kaplan-Meier survival curves produced by the R package “Survminer”.

### Developing a nomogram and its corresponding calibration curve

A nomogram is established based on clinical characteristics combined with risk score by R “rms” package. Calibration curves are created to determine the deviation between predicted and actual survival status.

### Gene set enrichment analysis (GSEA)

An analysis of GSEA is conducted in order to detect enriched biological pathways in low- and high-risk groups, respectively. (GSEA 4.2.1.).

### Immune cell infiltration analysis

“Estimation of STromal and Immune cells in MAlignant Tumor tissues (ESTIMATE) algorithm” is used to evaluate proportion of immune and stromal cells of each sample. Survival curves were plotted via the Kaplan-Meier method to predict the prognosis of COAD patients in the low- and high-stromal/immune score groups. Moreover, “Cell-type Identification by Estimating Relative Subsets of RNA Transcripts (CIBERSORT)” are performed to calculate the relative proportion of 22 immune cell infiltration of COAD samples from the training set. Then, the results calculated by CIBERSORT were screened according to *p*-value <0.05. The relationship between risk scores and different types of immune cells of each sample was evaluated via R “corrplot” package.

### Immune checkpoints analysis

Spearman correlation test is performed to determine the relationship between immune checkpoint molecules and risk scores.

### Prediction of drug therapy response

The drug-response prediction was evaluated with the R pRRophetic package and 2D conformations of drugs were visualized by PubChem website.

### Cell culture

HCT116 and DLD-1 cells are derived from ATCC. In this experiment, cells are cultured with RPMI-1640 medium supplemented with L-glutamine and 10% fetal bovine serum.

### Western blotting

WFS1 antibody (11558-1-AP) is purchased from Proteintech. The protein level of WFS1 in cells was measured through typical western blotting process. Beta-actin served as a loading control for western samples. By using ECL reagent, the protein concentration of WFS1 and beta-actin is determined. Two independent siRNAs are employed to decrease the WFS1 expression. The sequences of these two siRNAs used in this study are indicated as follows: si-WFS1-1: 5′-GCA GCG AGU CCA AGA ACU ACA-3′, si-WFS1-2: 5′-GCG UGA CUG ACA UCG ACA ACA-3′.

### Cell viability assay

HCT116 and DLD-1 cells are transfected with siRNAs targeting WFS1 or scrambled in 6-well plate. After 24 hours transfection, we seeded 4000 cells per well in 96-well cell culture plates. The OD value at 450 nm is measured at indicated time points via a CCK8 kit.

### Clone formation tests

HCT116 and DLD-1 cells are transfected with siRNAs targeting WFS1 or scrambled in 6-well plate. After 48 hours transfection, we seeded 500 cells per well in 6-well cell culture plate. After 2 weeks culture, the cells are stained with crystal violet.

### EdU assay

HCT116 and DLD-1 cells were seeded in 24-well plates at about 50% in confluency. In cells with 70% cell density, cells were transfected with scrambled or two independent siRNA targeting WFS1. After 48 hours culture, cells were incubated with EdU for 2 hours. Next, a solution of 4% paraformaldehyde was used to fix the cells. According to the manufacturer's instructions, the staining process was performed. Images were obtained through Nikon microscope and the ratio of EdU positive cells was measured via the ImageJ software.

### Consent for publication

All authors consent to the publication of this study.

### Availability of data and material

All data and R script in this study are available from the corresponding author upon reasonable request. Publicly available datasets were analyzed in this study, these can be found in The Cancer Genome Atlas (https://portal.gdc.cancer.gov/) and Gene Expression Omnibus (GSE40967). All authors read and approved the final manuscript.

## RESULTS

### Identify differentially expressed ERSRGs signature

**Figure 1:**
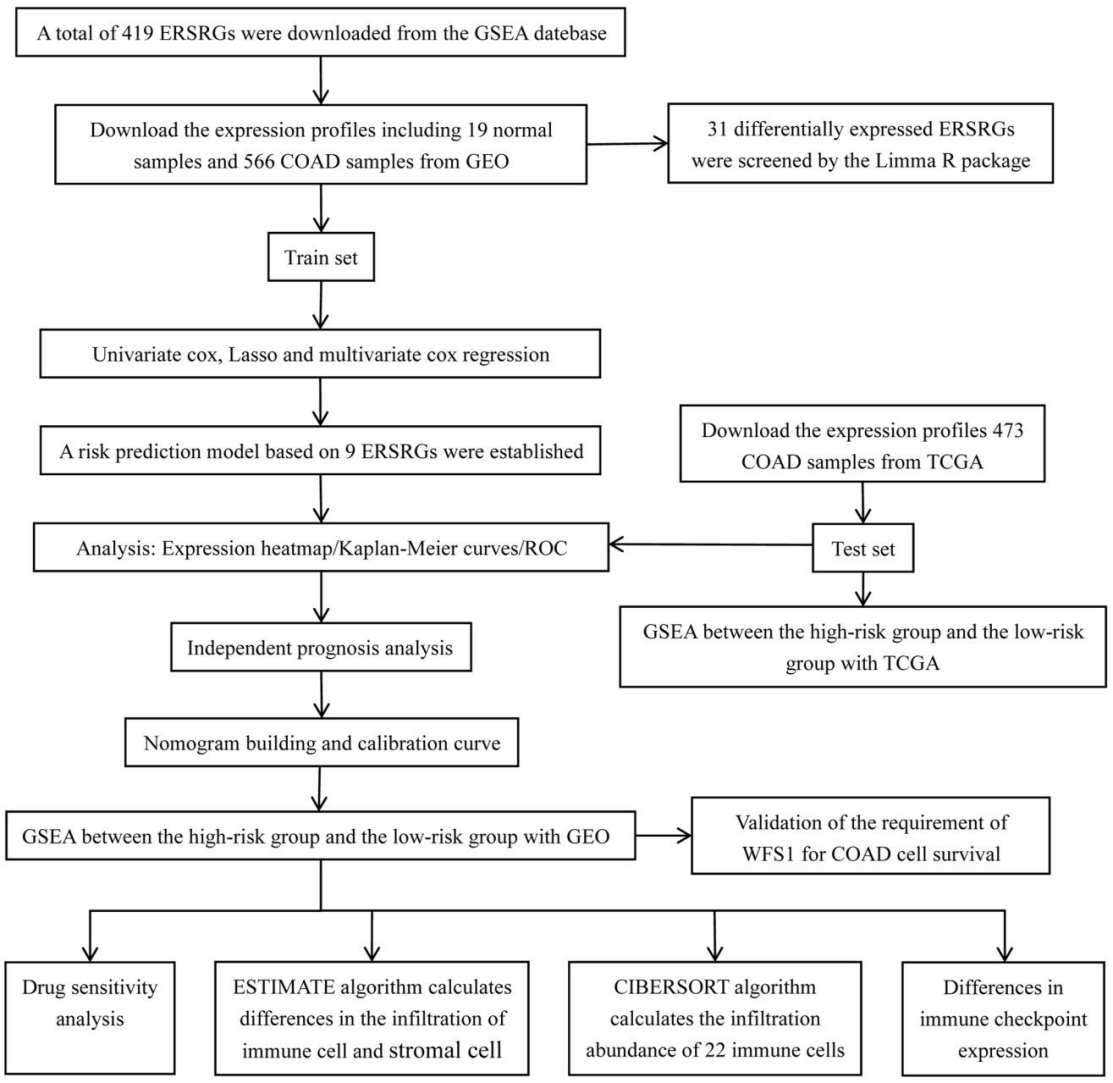
Flow chart of experimental design in this study.

The overall workflow of this study is depicted in Figure 1. According to the screening criteria of | log2FC | >1 and adjust *P* < 0.05, we identified 31 DEGs between COAD samples and colon normal tissues in the training set. Within COAD samples, 21 ERSRGs were dramatically down-regulated and 10 up-regulated compared to normal colon tissue (Supplementary Figure 1A–1C). A PPI network was obtained through uploading these 31 differentially expressed ERSRGs to STRING website (Supplementary Figure 1D), and CXCL8, CCND1, PSAT1, SLC7A5, EIF4EBP1, DDIT4, TRIB3, RRP9, NOLC1, and CEBPB were determined as hub genes with top ten scores (Supplementary Figure 1E).

### Construction of a prognostic model relied on ERSRGs

**Figure 2:**
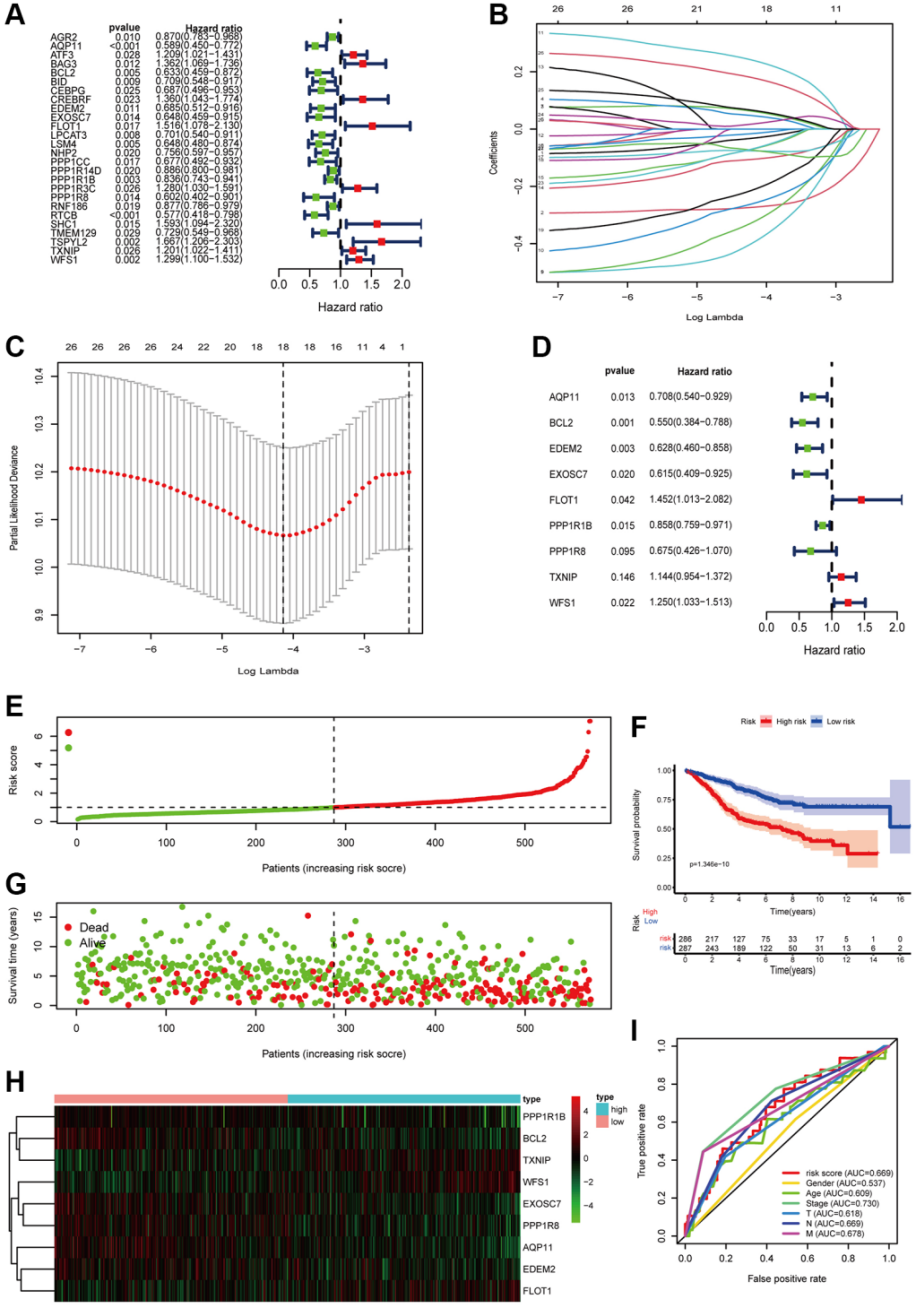
**Construction of risk model in the GSE40967 cohort.** (**A**) Univariate Cox regression analysis of ERSRGs associated with OS of COAD patients. (**B**) Lasso regression analysis of the ERSRGs based on univariate Cox regression analysis. (**C**) Cross-validation for tuning the parameter selection in the LASSO regression. (**D**) Multivariate cox regression analysis of the ERSRGs based on LASSO regression analysis. (**E**) Distribution of patients based on the risk score. (**F**) Kaplan-Meier curve of survival probability of patients in the high-risk group and low-risk group. Statistical tests were performed using the Chi-square test with statistical significance at *P* < 0.05. (**G**) Survival time and survival status of patients with different risk scores. (**H**) The heatmap of the expression of prognostic ERSRGs between high-risk group and low-risk group. (**I**) ROC curve of risk score and other indicators.

First, 26 ERSRGs were screened out via univariate Cox regression analysis univariate using the cutoff of *P* < 0.05, which were associated with overall survival of patients with COAD in the training set, including 9 risky genes and 17 protective genes (Figure 2A). In order to further shrink the variables, LASSO regression analysis was conducted based on these 26 ERSRGs (Figure 2B, 2C). Finally, 9 ERSRGs were determined to construct the prognostic model according to the multivariate Cox regression, including 3 risky genes and 6 protective genes (Figure 2D). The formula to calculate the risk score is indicated as follows: Risk score = (−0.34508 × AQP11) + (−0.5975 × BCL2) + (−0.46549 × EDEM2) + (−0.48618 × EXOSC7) + (0.373054 × FLOT1) + (−0.15286 × PPP1R1B) + (−0.39254 × PPP1R8) + (0.134627 × TXNIP) + (0.223501 × WFS1). COAD patients in training set were classified into high-risk and low-risk groups according to the median risk scores (Figure 2E). COAD patients in the high-risk group had worse prognosis compared to those in the low-risk group (Figure 2G). The heatmap (Figure 2H) presented expression levels of 9 ERSRGs in different groups. Kaplan-Meier survival curves suggested that COAD patients in the low-risk group had significantly better prognosis than those in the high-risk group (Figure 2F). The area under the receiver operating characteristic (AUC) curve was 0.669, showing that prognostic model we built had a good predictive performance (Figure 2I). Next, we detected whether the expression levels of these 9 ERSRGs were associated with prognosis of patients with COAD. We found that the expressions of APQ11, EDEM2, EXOSC7, PPP1R8, TXNIP, and WFS1 were strongly correlated with the OS of COAD patients (*p* < 0.05) (Supplementary Figure 2A–2I).

### Validation of the prognostic model

**Figure 3:**
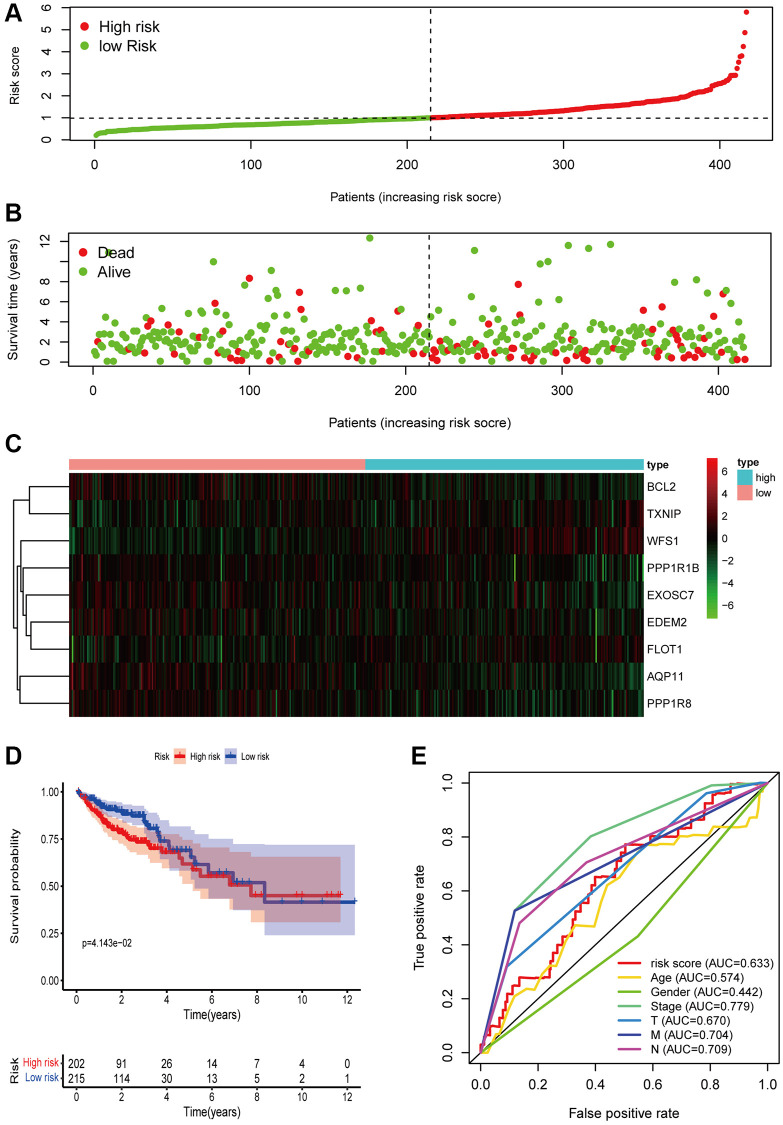
**Verification of risk signature in the TCGA cohort.** (**A**) The distribution of patients based on the risk score. (**B**) Survival time and survival status of patients with different risk scores. (**C**) The heatmap of the expression of prognostic ERSRGs in the high-group and low-risk group (**D**) Kaplan-Meier curve of survival probability of patients in the high-risk group and low-risk group. Statistical tests were performed using the Chi-square test with statistical significance at *P* < 0.05. (**E**) ROC curve of risk score and other indicators.

To validate the performance of the prognostic model in independent cohort, we downloaded the gene expression files of patients with COAD from TCGA as the test set. The risk scores of patients from test set were calculated using the same formula. COAD patients from the test set were separated into high-risk (*n* = 202) and low-risk (*n* = 215) groups according to the median risk score of the training set as the cutoff point. (Figure 3A). Similar with patients in training set, those with high-risk scores had shorter OS than those with low-risk scores (Figure 3B). The heatmap (Figure 3C) showed expression levels of 9 ERSRGs of each patient in test set. Kaplan-Meier survival curves suggested that COAD patients in the low-risk group had dramatically better prognosis compared with those in the high-risk group (Figure 3D). Our prognostic model had a good predictive performance, with an area under the receiver operating characteristic curve of 0.633 (Figure 3E).

### Determination of independent prognostic factors

To confirm whether the risk score of the prognostic risk model has independent prediction ability, we evaluate the prognostic value of risk score and clinical features in the training set. Univariate Cox regression analysis revealed that risk score, age, stage, T, M, and N could be used to predict outcomes of COAD patients (Supplementary Figure 3A). Furthermore, multivariate Cox regression verified that risk score, age, T, M, and N could still be regarded as independent prognostic predictors (Supplementary Figure 3B). These data demonstrated that prognostic risk model, age, T, M, and N were independently correlated with the outcomes of COAD patients.

### Construction of the nomogram

To better predict outcomes of COAD patients, we built a nomogram relied on risk scores combined with clinical characteristics to assess the 1-, 3- and 5-year survival probability (Supplementary Figure 4A). The calibration curves verified that the predicted prognosis from the nomogram have good consistency with the actual outcomes, which indicated that nomogram has good prediction performance (Supplementary Figure 4B–4D).

### Gene set enrichment analysis (GSEA)

For the purpose of uncovering the biological pathways that differentiate patients of high-risk from low-risk group, we conducted GSEA on the expression data from GSE40967 and TCGA cohorts, respectively. The result of KEGG enrichment analysis revealed that Arrhythmogenic right ventricular cardiomyopathy, Citrate cycle, and Dilated cardiomyopathy were significantly enriched in high-risk group in GSE40967 cohort. Meanwhile, Huntington’s disease, Hypertrophic cardiomyopathy, Oxidative phosphorylation, Parkinson’s disease, and Pyruvate metabolism pathway were dramatically enriched in low-risk group in GSM40967 cohort (Supplementary Figure 5A). Moreover, genes from high-risk group in TCGA were enriched in Arrhythmogenic right ventricular cardiomyopathy, Cell cycle, Complement and coagulation cascade. However, Homologous recombination, Hypertrophic cardiomyopathy, Purine metabolism, Pyrimidine metabolism, and RNA degradation pathway were abundant among low-risk group in TCGA (Supplementary Figure 5B).

### Identifying the differences of infiltration of immune cells

Next, we performed tumor microenvironment analysis using ESTIMATE algorithm and evaluated the proportion of immune cell infiltrated in tumor tissue using CIBERSORT. Stromal scores of patients in the high-risk group were significantly higher than those in the low-risk group in the training set (Supplementary Figure 6A). Accordingly, COAD patients in the high-stromal score group had worse prognosis compared to those in low-stromal score group (Supplementary Figure 6B). In addition, the infiltration of B cells memory, Plasma cells, T cells CD4 memory activated, and Neutrophils had statistical difference between high-risk and low-risk groups, respectively (Supplementary Figure 6C). Scatter plots (Supplementary Figure 6D–6H) showed that risk scores were positively associated with infiltration levels of B cells memory, Neutrophils, and Mast cells activated (R > 0, *P* < 0.05), and negatively associated with infiltration levels of Plasma cells, and T cells CD4 memory activated in the immune microenvironment (R < 0, *P* < 0.05).

### Identifying the different expression level of immune checkpoint genes

The expressions level of CD27, CD48, HHLA2, ICOSLG, IDO1, NRP1, TNFRSF14, TNFRSF18, TNFRSF9, TNFSF14, TNFSF4, and TNFSF9 had statistical difference between high-risk and low-risk groups, especially for NRP1, TNFRSF14, and TNFSF4 (*P* < 0.001) (Supplementary Figure 7A). The Spearman correlation analysis indicated CD27 was markedly positively associated with CD48, IDO1, and TNFRSF9. Furthermore, CD48 was dramatically positively associated with DO1 and TNFRSF9. In addition, TNFRSF9 was positively correlated with IDO1. TNFSF4 was positively associated with NRP1 (Supplementary Figure 7B).

### Response of patients with COAD to candidate drugs

We estimated the response of COAD patients in these two groups to candidate drugs. IC50 values of 60 compounds were significantly different in high-risk and the low-risk patients (Supplementary Table 1). We visualized the top eight drugs with the greatest difference in *P* values. AZD.0530, Bryostatin.1, CHIR.99021, Imatinib, LFM.A13, and CCT007093 were more sensitive to high-risk compared with low-risk patients (Supplementary Figure 8A–8F). However, PF.4708671 and EHT.1864 showed the opposite trend (Supplementary Figure 8G, 8H).

### Knockdown of WFS1 molecule inhibits HCT116 and DLD-1 cells growth

**Figure 4:**
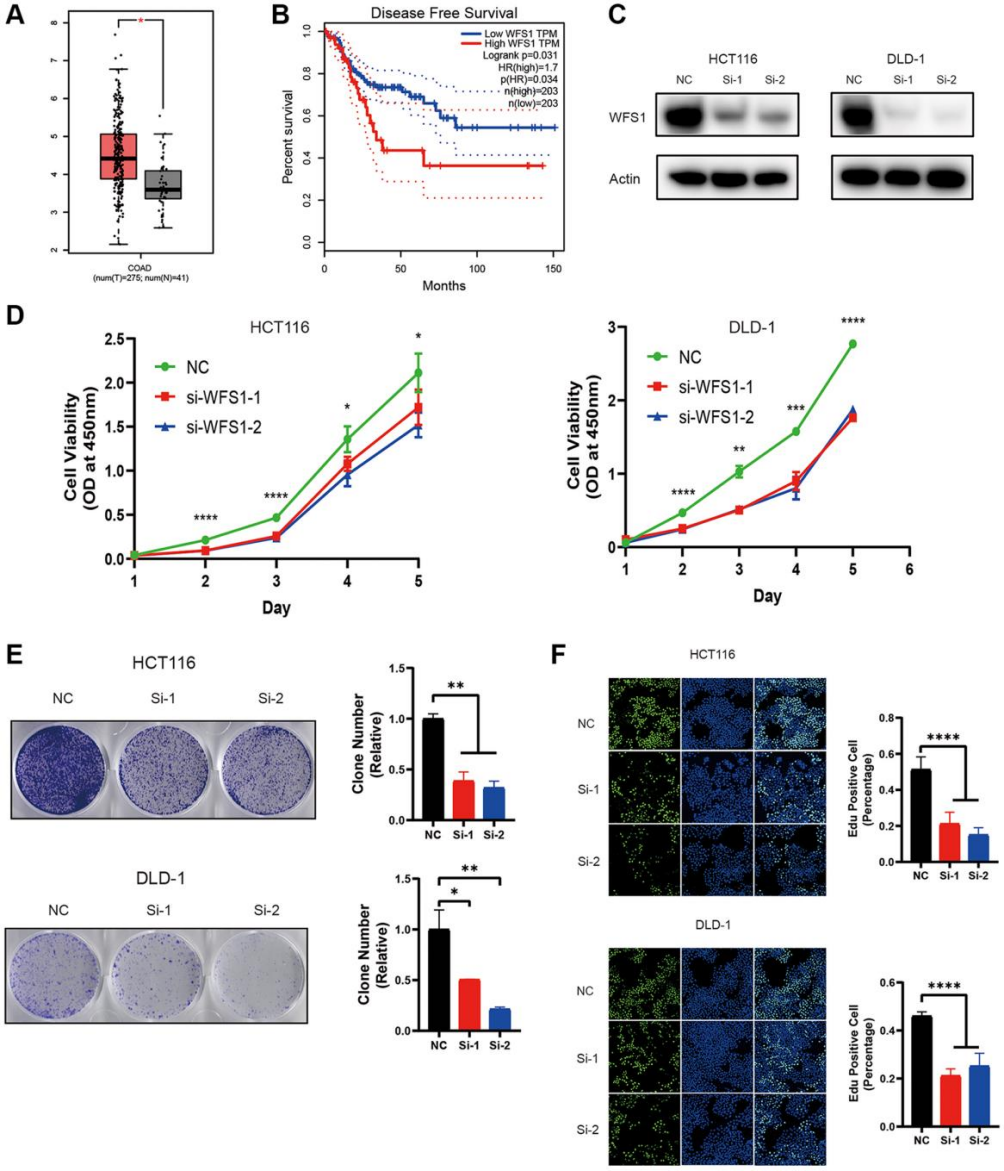
**Knockdown of WFS1 inhibits COAD cell proliferation.** (**A**) Box plots showed that WFS1 expression was significantly higher in COAD than in normal samples. (**B**) Survival curves showed that patients in the low WFS1 expression group had a better prognosis than those in the high WFS1 expression group. (**C**) Western blot analysis of WFS1 expression in HCT116 and DLD-1 cell lines. actin is internal control. (**D**) Cell viability of HCT116 or DLD-1 cells transfected with control or WFS1siRNAs was measured by CCK8 assay. (**E**) Clone formation assay of HCT116 or DLD-1 cells transfected with control or WFS1siRNAs. (**F**) EdU staining for cell proliferation transfected with control or WFS1siRNAs.

We investigated the WFS1 expression in COAD and normal colon tissues in TCGA. Expression levels of WFS1 in COAD were dramatically higher than those in normal colon tissues (Figure 4A). Accordingly, patients with lower WFS1 expression had a longer OS (Figure 4B). These findings indicate that WFS1 has the potential to be a promising target for the therapy of colon cancer.

In order to evaluate the potential role of WFS1 in COAD, we inhibited the expression of WFS1 with two independent siRNAs in HCT116 and DLD-1 cell lines. These two siRNAs significantly decrease the protein expression of WFS1 (Figure 4C). WFS1 depletion markedly inhibited cell viability and clone formation capability of COAD cells (*P* < 0.05, Figure 4D–4F). In summary, these results indicate that WFS1 has a potential carcinogenic effect in COAD.

## DISCUSSION

According to the statistics from World Health Organization, amount of newly diagnosed COAD patients worldwide in 2020 was 1,148,515, accounting for 6.0% of the global new cancer patients. The global colon cancer deaths totaled 576,858 in 2020, accounting for 5.8% of the global cancer deaths [12]. At present, the treatment of colon cancer patients mainly includes surgical resection, chemotherapy, and radiotherapy. However, the specific clinical symptoms of early colon cancer patients are not obvious. Patients with COAD are more likely to be diagnosed in the late stage. Most of the COAD patients have a tendency to recurrence after surgery. Thus, novel biomarkers are highly needed to estimate outcomes of COAD patients.

Protein processing, modification, and folding in ER are closely regulated processes that determine cell function, destiny, and survival [10]. Adjustment of tumor growth and anti-tumor immunity by UPR is a prospective opportunity for cancer therapy [13]. A large number of studies indicated that ER stress with certain intensity promotes the tumor progression, migration, therapeutic resistance, and angiogenesis [14]. Numerous studies have shown that growth inhibition of COAD cells by many compounds is associated with the induction of endoplasmic reticulum responses [15–20].

In this study, we identified nine ERSRGs (AQP11, BCL2, EDEM2, EXOSC7, FLOT1, PPP1R1B, PPP1R8, TXNIP, WFS1) associated with OS and constructed a prognostic model. Survival probability of COAD patients in the high-risk group was significantly lower than those in the low-risk group. In addition, multivariate regression proved that risk score can served as an independent prognostic factor. Subsequently, a nomogram relied on clinical characteristics and risk score was established to quantitatively predict survival rates of COAD patients. Furthermore, the GSEA analysis found that ERSRGs were associated with several metabolism and cancer related pathways. These results suggest that these nine ERSRGs may be new targets for COAD treatment.

The level of immune cell infiltrated in tumor significantly determined the prognosis of COAD patients [21]. Previous study showed that T cells, B cells and natural killer (NK) cells in TME were highly correlated with prognosis in COAD patients [22]. COAD antigen is able to induce antitumor response which is mediated by CD4^+^ T cells [23]. In patients with solid tumors, neutrophils expansion in TME is usually correlated with unfavorable prognosis [24]. Our study supports these findings. Moreover, immunosuppressive molecules such as CD27, CD48, HHLA2, ICOSLG, IDO1, TNFRSF14, TNFRSF18, and TNFRSF9 in the low-risk group were markedly higher compared to those in the high-risk group, demonstrating that patients with low expression level of immunosuppressive molecule were more likely to benefit from immune checkpoint blockade (ICB) therapy.

Next, we identified that IC50 values of 60 compounds were different in the high-risk and the low-risk patients. Patients from high-risk group were more sensitive to AZD.0530, Bryostatin.1, CHIR.99021, Imatinib, LFM.A13, and CCT007093 than those in low-risk group, while low-risk patients were more likely to benefit from PF.4708671, and EHT.1864. In the previous studies, Bryostatin-1 is a macrolide derived from marine invertebrates that could suppress the terminal differentiation of colon cancer cells by downregulating PKC-mediated proteoglycan metabolic pathway [25]. Imatinib, a 2-phenylaminopyrimidine derivative, inhibits the proliferation of COAD cells [26]. LFM-A13 is the active metabolite of leflunomide that suppresses COAD cell growth [27]. However, the mechanism of the compounds inhibiting the progression of COAD requires further research.

Consistent with previous research, these prognostic ERSRGs play diverse roles in different cancers. Consistent with previous studies, BCL2 is upregulated in breast, prostate, colorectal, lung, stomach, ovarian cancer, and other solid tumors [28]. The inhibition of BCL2 expression by hsa-miR-139-5p in colorectal cancer cells increased chemosensitivity [29]. Chuyong Lin et al. confirmed that FLOT1 promotes breast cancer cell proliferation and tumorigenesis [30]. Ferreira et al. indicated that PPP1R8 is crucial for the maintenance of the male germline and spermatogenesis [31]. Chow, Pak Hin et al. suggested that increasing levels of AQP11 were associated with better survival rates in colorectal and breast cancers [32]. Some studies demonstrated that PPP1R1B was overexpressed in diverse human cancers, including colon, breast, and gastric cancer. And PPP1R1B may also regulate pro-oncogenic signal transduction pathways to promote chemoresistance and increase gastric and breast cancer cell viability [33, 34]. It is reported that TXNIP is a tumor suppressor, which has been verified in various cancers, including breast, lung, and thyroid cancer. What’s more, it can also inhibit tumor cell growth and induce apoptosis and cell cycle arrest [35]. WFS1 autosomal recessive deletion mutation can lead to Wolfram syndrome. Yamada found that WFS1 deficiency increased ER stress, impaired the cell cycle, and ultimately promoted pancreatic β cell apoptosis [36]. The role of EDEM2 in cancer remains unclear. Interestingly, Weilong Zhang et al. found that EXOSC7 is a risky gene in patients with mantle cell lymphoma, which is inconsistent with our findings [37]. This may be due to different genetic backgrounds of different types of cancer, or to different selection of data sets. Moreover, by knocking down the prognostic differential gene WFS1 in HCT116 and DLD-1 cell lines with two siRNAs, we found that WFS1 had a potential carcinogenic effect in COAD.

## CONCLUSIONS

Based on 9 ERSRGs (AQP11, BCL2, EDEM2, EXOSC7, FLOT1, PPP1R1B, PPP1R8, TXNIP, WFS1), we successfully constructed a prognostic risk model that precisely estimate the prognosis of COAD patients. Our findings may offer novel targets for the therapy of COAD patients.

## Supplementary Materials

Supplementary Figures

Supplementary Table 1
