# Supplementary material for: System analysis based on the ER stress-related genes identifies WFS1 as a novel therapy target for colon cancer
Source: Aging (Albany NY). 2022 Nov 28;14(22):9243–63. doi: 10.18632/aging.204404 (PMC9740360; doi:10.18632/aging.204404)
Supplement: Supplementary Figures [file aging-14-204404-s001.pdf]

SUPPLEMENTARY FIGURES

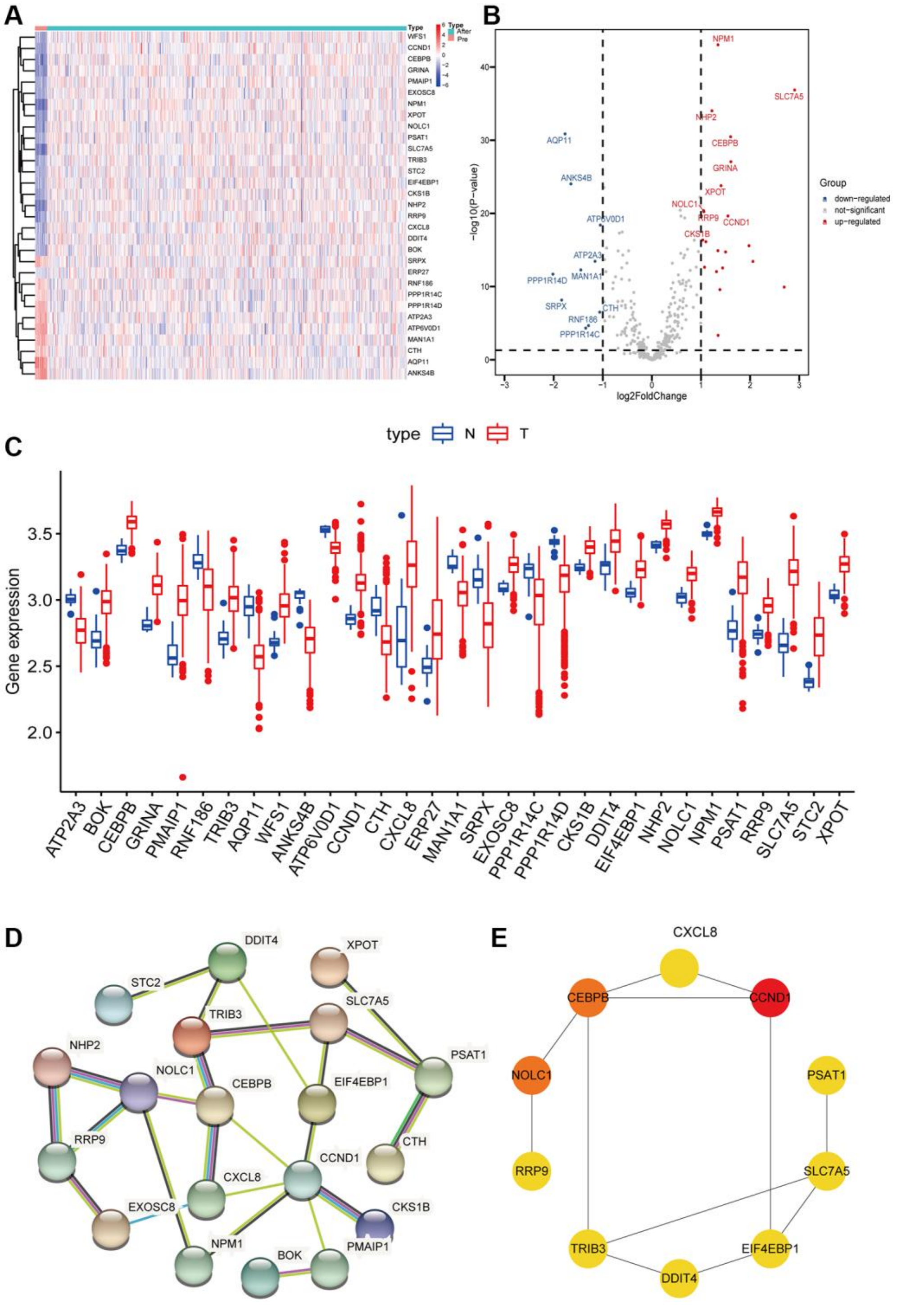

Supplementary Figure 1. The differentially expressed ERSRGs between colon cancer and normal samples. (**A**) Heat map showing expression levels of genes between COAD samples and normal samples. Volcano plot (**B**), and boxplot (**C**) describe differentially expressed genes between COAD samples and normal samples, red dots represent significantly up-regulated genes, blue dots represent significantly down-regulated genes, and grey dots represent genes with no difference. (**D**) Construction of protein-protein interaction (PPI) network of differentially expressed genes using the STRING database. (**E**) The hub genes map was drawn using the MCC algorithm in Cytoscape software.

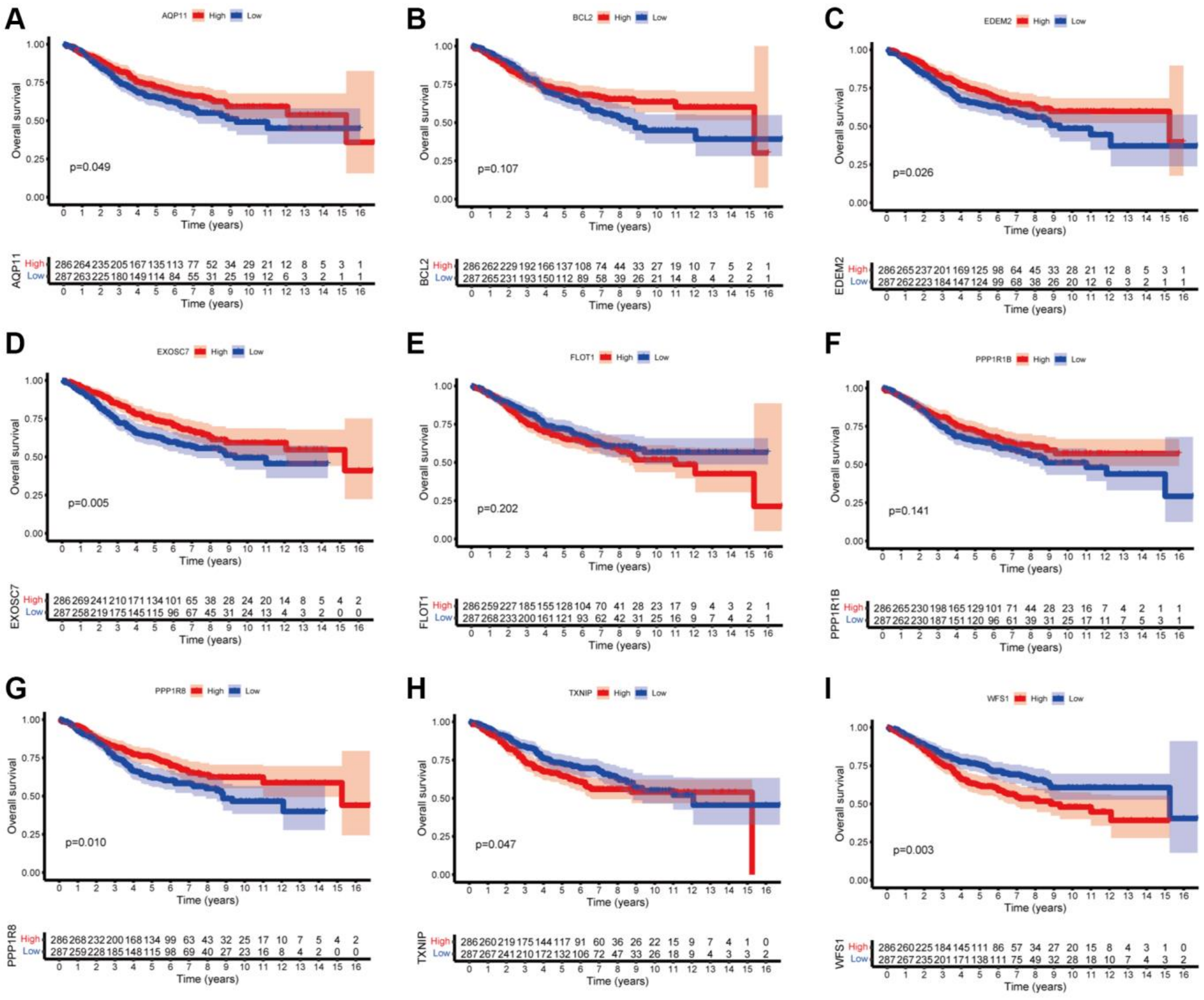

Supplementary Figure 2. Kaplan-Meier survival curves of prognostic ERSRGs in GSE40967 dataset, including (**A**) AQP11, (**B**) BCL2, (**C**) EDEM2, (**D**) EXOSC7, (**E**) FLOT1, (**F**) PPP1R1B, (**G**) PPP1RB, (**H**) TXNIP, (**I**) WFS1. Statistical tests were performed using the Chi-square test with statistical significance at $P < 0.05$.

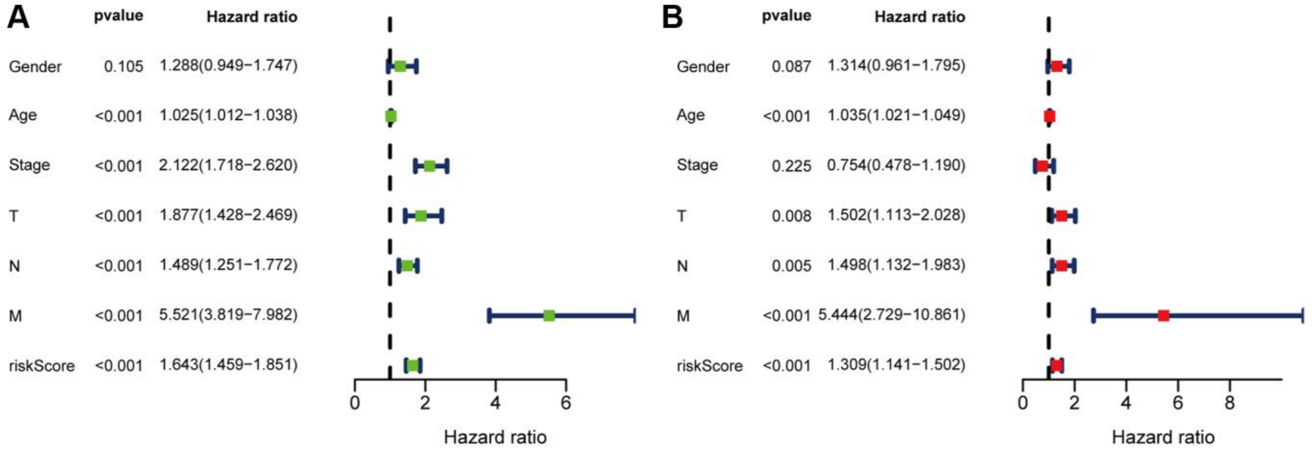

Supplementary Figure 3. Univariate and multivariate Cox regression analysis of risk score and clinical characteristics in the GEO cohort. (**A**) Univariate Cox regression results. (**B**) Multivariate Cox regression results.

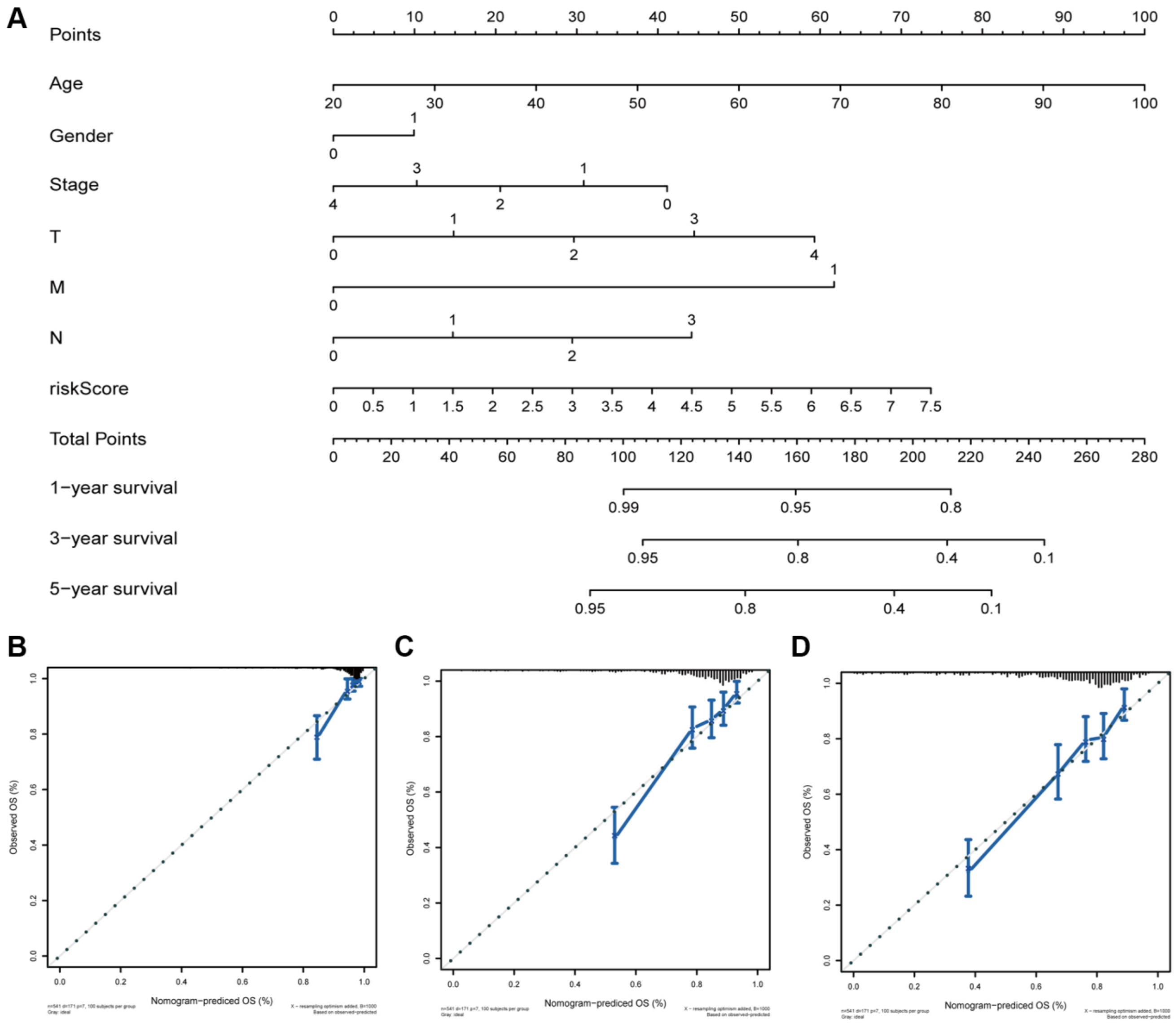

Supplementary Figure 4. Nomogram of predicting overall survival in COAD patients in the GEO cohort. (**A**) Nomogram based on risk score and Clinical features. (**B**–**D**) Calibration curves of predicting patient survival probabilities in 1-, 3-, and 5- years.

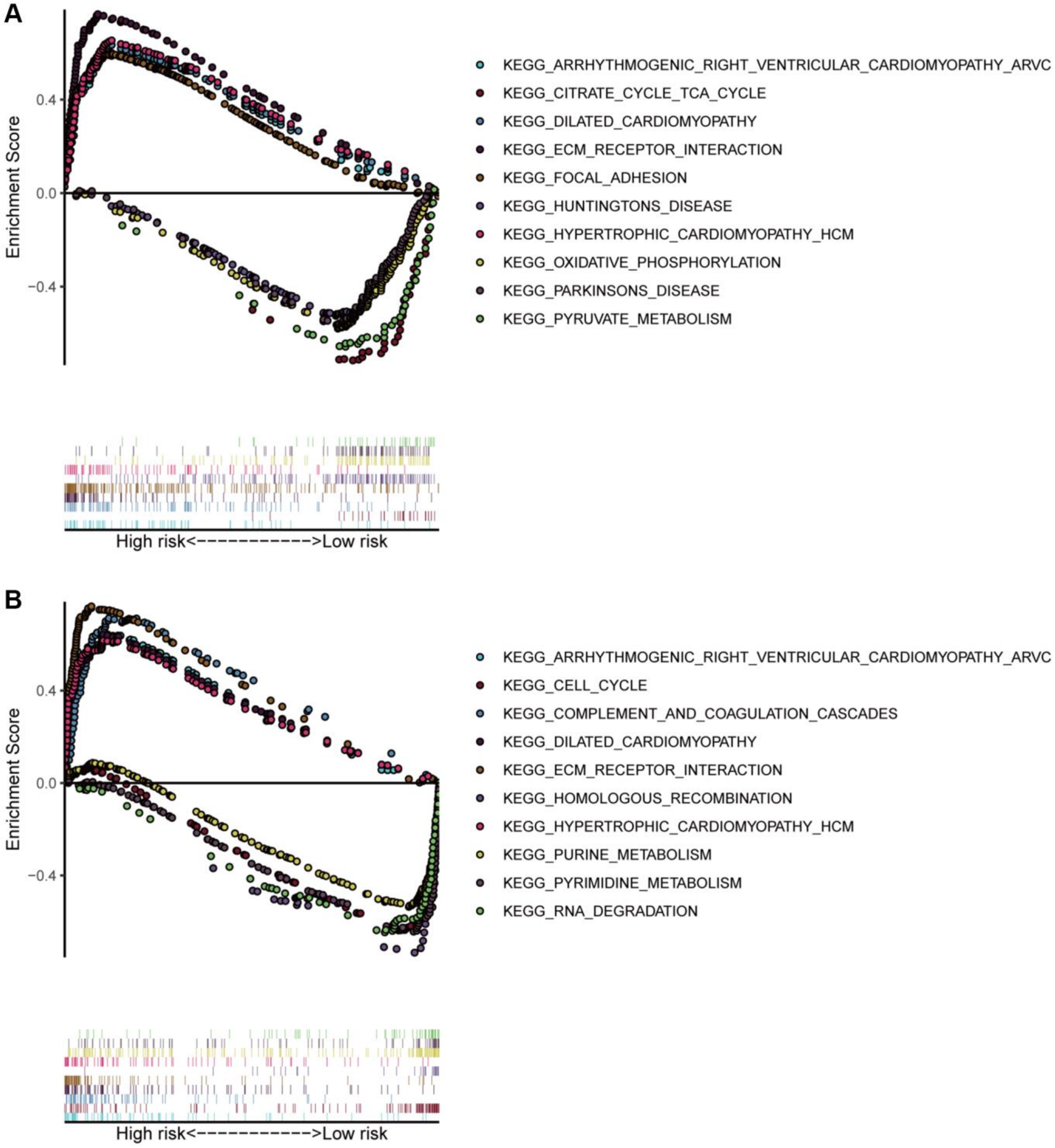

Supplementary Figure 5. GSEA analysis of training and test sets. (**A**) GSEA analysis of ERSRGs the between high- risk and low-risk groups in the training set. (**B**) GSEA analysis of ERSRGs between high- risk and low-risk groups in the test set.

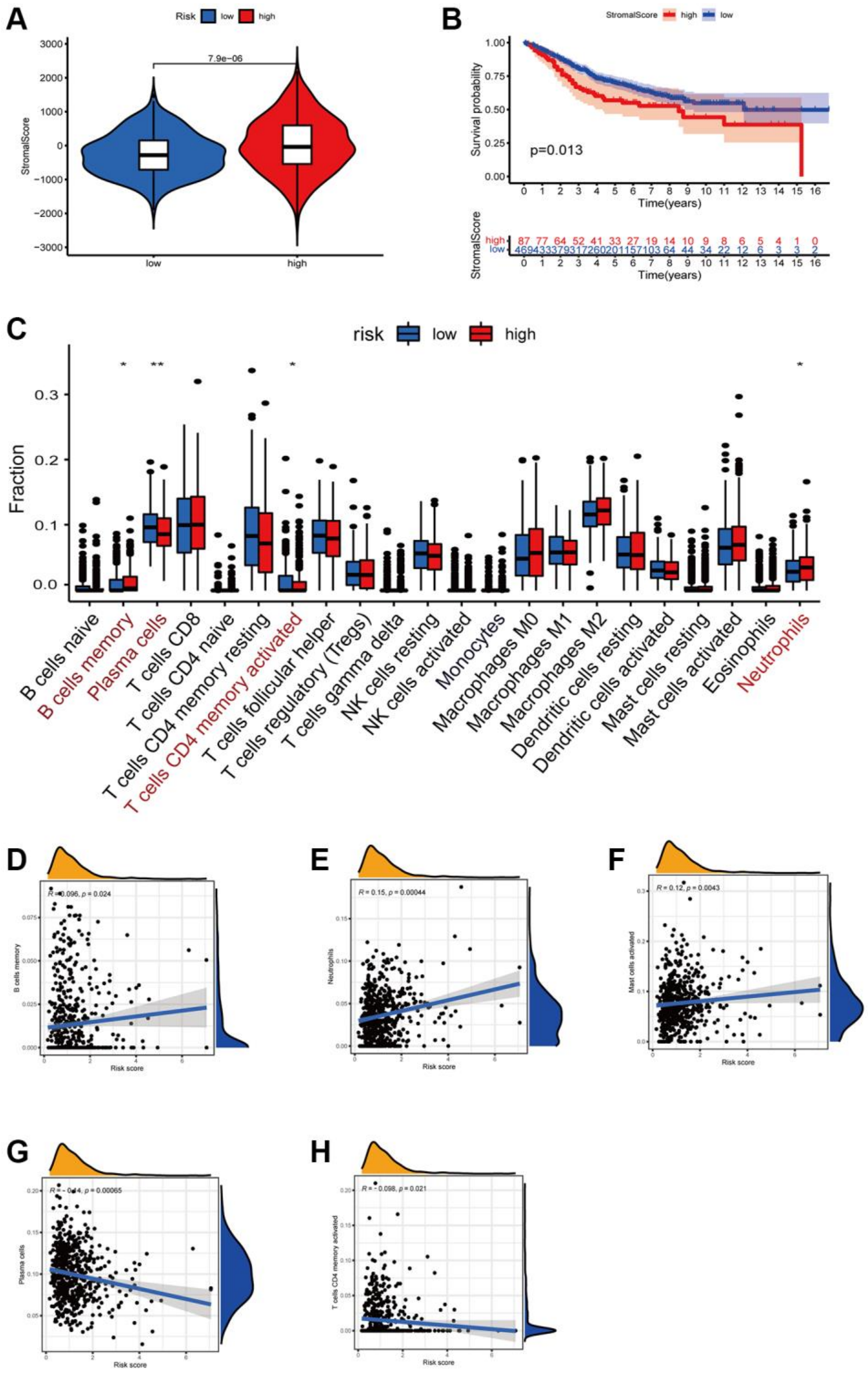

Supplementary Figure 6. Evaluation of immune cell infiltration in the high-risk group and low-risk group. (**A**) Violin plot of stromal score between high-risk group and low-risk group. (**B**) Kaplan-Meier survival analysis of stromal score. (**C**) Boxplot of infiltrated immune cells between high-risk and low-risk groups. (**D**–**H**) Correlation between risk score and immune cell infiltration.

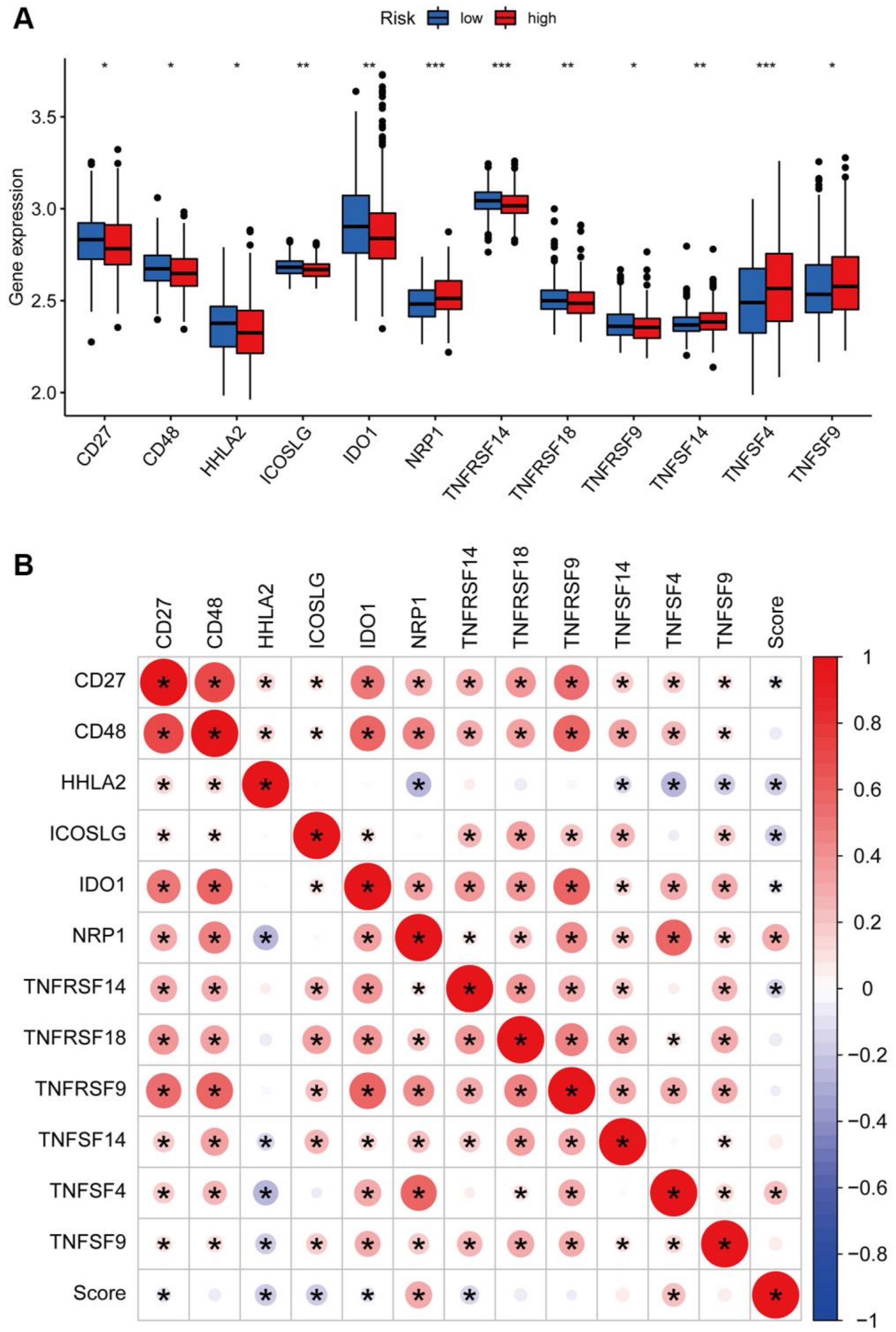

Supplementary Figure 7. Correlation of immune checkpoints and risk score. (**A**) The expression of immune checkpoint molecules between the high-risk and low-risk groups. (**B**) Spearman correlation analysis of immune checkpoints and risk score. Red represents positive correlation and blue represents negative correlation.

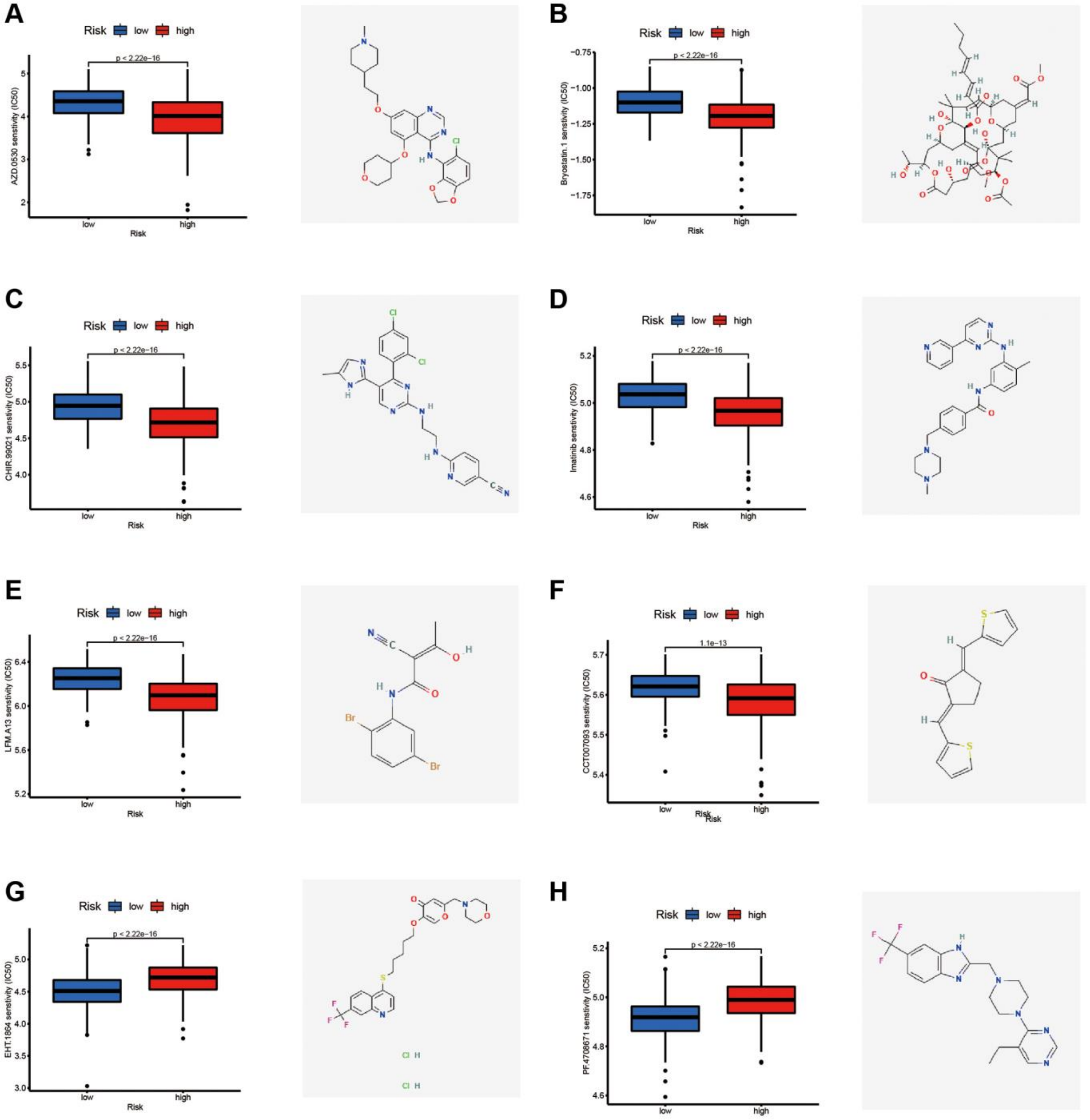

Supplementary Figure 8. The eight compounds with the most significant differences in susceptibility between the high-risk and low-risk groups as well as their 2D conformations. The IC50 values and corresponding 2D conformations of (**A**) AZD.0530, (**B**) Bryostatin.1, (**C**) CHIR.99021, (**D**) Imatinib, (**E**) LFM.A13, (**F**) CCT007093, (**G**) EHT.1864, and (**H**) PF.4708671.
