# Supplementary material for: System analysis based on the ER stress-related genes identifies WFS1 as a novel therapy target for colon cancer
Source: Aging (Albany NY). 2022 Nov 28;14(22):9243–63. doi: 10.18632/aging.204404 (PMC9740360; doi:10.18632/aging.204404)
Supplement: Supplementary Table 1 [file aging-14-204404-s002.pdf]

SUPPLEMENTARY TABLE

Supplementary Table 1. The 60 compounds with the differences in sensitivity between high- risk and low-risk groups.

Drug name	*P*-value
A.443654	2.10E-11
A.770041	8.50E-08
ABT.888	3.40E-06
AP.24534	4.10E-07
AS601245	4.70E-08
ATRA	2.50E-06
AZD.0530	2.22E-16
AZD6482	1.50E-05
Bexarotene	9.80E-06
BIBW2992	2.20E-05
Bicalutamide	7.60E-06
BMS.754807	2.20E-09
Bryostatin.1	2.22E-16
BX.795	7.40E-05
CCT007093	1.10E-13
CCT018159	2.90E-07
CHIR.99021	2.22E-16
Cytarabine	2.60E-05
Dasatinib	3.90E-11
DMOG	6.50E-10
EHT.1864	2.22E-16
Epothilone.B	5.50E-07
FTI.277	1.70E-12
GDC0941	1.20E-05
GNF.2	1.50E-06
GSK.650394	1.10E-05
GSK269962A	6.40E-07
GW843682X	9.40E-12
Imatinib	2.22E-16
JW.7.52.1	1.00E-07
LFM.A13	2.22E-16
Metformin	1.40E-07
Midostaurin	4.90E-08
NVP.TAE684	7.80E-05
OSI.906	2.50E-13
PAC.1	7.60E-06
Paclitaxel	8.30E-07
Parthenolide	2.30E-12
Pazopanib	2.60E-05
PF.562271	7.80E-05

PF.4708671	2.22E-16
PHA.665752	5.70E-08
PLX4720	1.40E-08
QS11	6.30E-07
Rapamycin	6.20E-09
RDEA119	1.80E-05
RO.3306	2.90E-06
S.Trityl.L.cysteine	2.30E-09
Salubrinal	1.10E-07
SB.216763	9.20E-09
SB590885	7.20E-05
SL.0101.1	2.70E-06
Sorafenib	1.50E-13
Tipifarnib	8.40E-07
Vinblastine	1.20E-05
Vorinostat	3.40E-08
VX.680	2.40E-10
VX.702	6.60E-08
WH.4.023	1.60E-08
WZ.1.84	3.40E-09
